# Qualitative assessment of innovations in healthcare provision

**DOI:** 10.1186/1472-6963-9-50

**Published:** 2009-03-19

**Authors:** Franz Porzsolt, Amit K Ghosh, Robert M Kaplan

**Affiliations:** 1 Clinical Economics, University of Ulm, Frauensteige 6, 89075 Ulm, Germany; 2 Department of Internal Medicine, Mayo Clinic, 200 1st Street SW, Rochester MN, 55905 USA; 3 Department of Health Services, UCLA School of Public Health, Los Angeles, CA 90025-1772, USA

## Abstract

**Background:**

The triad of quality, innovation and economic restraint is as important in health care as it is in the business world. There are many proposals for the assessment of quality and of economic restraints in health care but only a few address assessment of innovations. We propose a strategy and new structures to standardize the description of health care innovations and to quantify them.

**Discussion:**

Strategy and structure are based on the assumption that in the early phase of an innovation only data on the feasibility and possibly on the efficacy or effectiveness of an innovation can be expected. From the patient's perspective, benefit resulting from an innovation can be confirmed only in a later phase of development. Early indicators of patient's benefit will be surrogate parameters which correlate only weakly with the desired endpoints. After the innovation has been in use, there will be more evidence on correlations between surrogate parameters and the desired endpoints to provide evidence of the patient benefit. From an administrative perspective, this evidence can be considered in decisions about public financing. Different criteria are proposed for the assessment of innovations in prevention, diagnosis and therapy. For decisions on public financing a public fund for innovations may be helpful. Depending on the phase of innovation risk sharing models are proposed between manufacturers, private insurers and public funding.

**Summary:**

Potential for patient benefit is always uncertain during early stages of innovations. This uncertainty decreases with increasing information on the effects of the innovation. Information about an innovation can be quantified, categorized and integrated into rational economic decisions.

## Background

The triad of quality, innovation, and economic restraint is as important in the provision of healthcare as it is in the business world. In economics, an innovation refers not only to products (like therapies and diagnostic measures), but also to procedures and the development of new markets [1]. Transferring this understanding of innovation to a publicly-financed system is risky because forces that govern the free market are often not feasible in a publicly-financed system. We do not have a good understanding of how systems innovations can systematically, fairly, and transparently be transferred to healthcare provision. A Canadian proposal describes innovation in the healthcare system as the replacement of a previous method with a new approach that provides sustainable and competitive benefit. Benefit is sustainable and competitive if it keeps the organization economically healthy, introduces a rare or singular characteristic, and contains a learning process which enables the organization to achieve a leading position in the market [2].

According to the Veterans Affairs Health System, quality cannot be separated from innovation. Research results are immediately integrated into everyday clinical practice to either improve patient care or to increase the efficiency of the system [3]. Also the British National Institute for Health and Clinical Excellence (NICE) is confronted with a similar problem. It has to make well-balanced decisions among quality, innovation, and value for money [4]). In Germany we are in the stage of developing a similar system. A rapid transfer of innovation to the daily provision of healthcare must be combined with a critical test of its validity. If a new method does not fulfill expectations, a search for a better method to replace it is likely. However, these examples are exceptions. In many countries practices, once introduced, may get continuing application without ongoing evaluation. In the United States as well as in Germany, the Food and Drug Administration (FDA) in the U.S. and the corresponding authority in Germany requires that pharmaceutical firms maintain phase IV monitoring studies. However, the concern in these evaluations is in both countries surveillance for harm, rather than continuing evaluation of efficacy. Lack of efficacy may be considered as economic harm.

Access to provision of healthcare innovations is ultimately regulated by reimbursement policies. This dichotomous decision to fund or not fund a particular innovation is problematic because of the benefit of the intervention is often uncertain. Such uncertainty creates two risks in these decisions – either healthcare services of little benefit are publicly financed or access to highly beneficial healthcare services is denied. It is difficult to exclude publicly-financed services which have been supported and promoting untested innovations is associated with far-reaching risks. We believe it is necessary to have regulation which minimizes both risks, the undesired financing of services of little benefit and the undesired curbing of innovations. Uncoupling consumers from medical progress and loss of the stimulating effect on competition are among the far-reaching risks of curbing innovation.

To reduce uncertainty in decision-making about public support of innovative treatments, standardized cost-benefit and cost-utility assessments of healthcare services should be required whenever possible. The methodical drawbacks of these procedures are well known, but are accepted because they still improve the decision process. Since this discussion, including the value assessment of new medications, is ongoing in Germany, many parties have suggested accepting the internationally recognized standard (with its known deficiencies) while simultaneously working on improved acceptable solutions [5,6].

Comparing innovations is challenging because the benefit of different innovations cannot be evaluated with identical criteria. The evaluation of preventive measures [7] and of diagnostic measures [8] requires considerations which are too complex and too far removed from an acceptable solution to be applied in a standardized way. These limitations should be taken into consideration to avoid overestimating the practical relevance of criteria which we apply to assess innovations. We illustrate this with examples from prevention and diagnosis.

A preventive measure will be considered valuable if the achievement of the desired endpoint (like avoiding late consequences of a metabolic syndrome) can actually be confirmed. The benefit will be valued less highly if it can be proved that surrogate markers closely correlated with the endpoint (like a significant reduction in body mass index) is attained and maintained. If, however, there are no data describing the chances or attaining the endpoint, the benefit of this innovation will be considered small.

Diagnostic measures also require a discriminating evaluation because the benefit to the patient deriving from the diagnosis depends on a successful intervention for the problem that has been diagnosed. A number of hurdles lie between a correct diagnosis and a successful resolution of the problem in everyday clinical practice, like deducing the correct diagnostic result, correctly managing the accompanying illnesses, and achieving high patient compliance. In everyday practice it is rarely recognized that each of these hurdles must be successfully overcome to reach the desired goal. Intermediate goals are extremely important, but are not sufficient to serve as the basis of a benefit-oriented allocation of resources [9].

The solution may lie in a compromise – dividing the assessment of innovations into two parts, the evaluation of less and of more complex problems. Less complex problems could be evaluated by statements upon which agreement can be reached and which are considered to be supported by scientific theory and evidence. Complex problems cannot be solved by descriptions and explanations because there is no way to get the unequivocal evidence. They can only be solved by norms such as recommendations, guidelines or laws. These norms cannot be prescribed by scientists. Norms require deliberation beyond the scientific process and must include the public and policy makers. We refer to this process as democratic authorization. We as scientists shall herein restrict ourselves to suggestions for solving simpler problems, which can be supported by true, scientific statements.

We propose qualitative assessment of innovations in healthcare provision, although we are well aware that an evaluation of innovations, just as evaluation of the benefit of healthcare services, concerns normative, but not "true", statements and requires democratic authorization.

## Discussion

### Qualitative Instead of Dichotomous Assessment of Innovations

Clinical decisions on innovations are problematic because we are uncertain whether the assumed advantages will materialize and the expected disadvantages will remain within the set limits. The data that justify the decisions are often unavailable before most medicines are licensed.

The risk of making incorrect decisions can be reduced if the final decision can be delayed until relevant data are available and a provisional decision based on existing information is offered. The better the available data the less will be the risk. The decisive factor is information that is based on the results and not on structures or processes which, in the final analysis, describe the quality of patient care. This point of view, which contains the three components, is shared by Portuguese authors [10], e.g., in "Inovacão, Evidencia e Mondo Real" (Innovation, Evidence, and the Real World), which describes the innovative idea, the data which confirm its feasibility, and the result obtained under everyday conditions.

#### Public Agency

We propose that new public agencies are required to oversee the funding of innovations. Current strategies have serious limitations. For example, the United States Food and Drug Administration primarily focussed on safety. Although they do require some continuing monitoring of pharmaceutical products, the FDA does not provide enough information for the comparison of relative effectiveness. Further, the FDA does not consider cost-effectiveness or many components of patient reported wellness.

One concern with contemporary approaches is that conflicts of interest are common. For example, members of committees that create guidelines often receive financial support from companies that make the products they are evaluating [11,12].

We propose national agencies similar to the National Institute for Health and Clinical Excellence (NICE). These agencies would focus on clinical effectiveness and would require specific components in their evaluation. The agencies would provide clinical information and outcomes study evaluations long after products are licensed. Components of the evaluations would include clinical outcome studies, evaluations of the relevance of the outcome measures, and cost-utility assessments. Based on these data the agency would also make prioritization recommendations but no effective decisions for publicly supported programs. The effective decisions about public health care services have to be made by an agency with democratic authorization. Guidelines would be available to private payers who could make their own decisions about whether or not to apply the guidelines.

The public agencies would draw on peer review panels of experts and would have very strict conflict of interest disclosure requirements.

#### Components of Decision Process Used by Agencies

**Figure 1:**
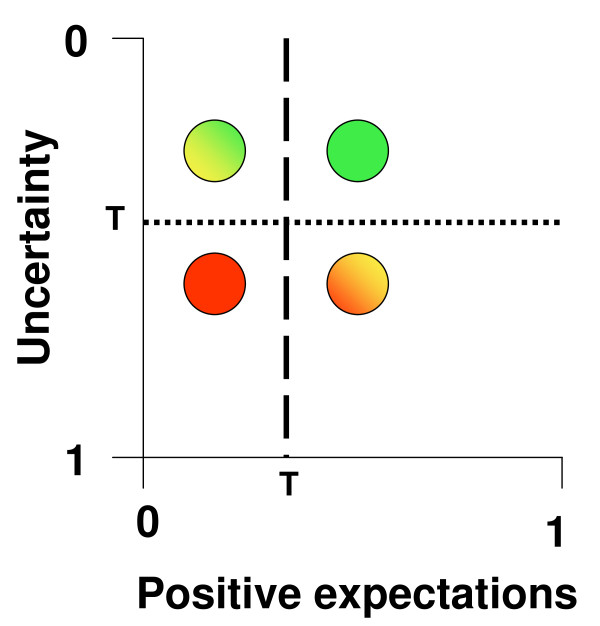
**The expectation-uncertainty-diagram of innovations**. Positive expectations in an innovation as well as uncertainty of data (as assessed by validity and variance of results) are rated on a scale ranging from zero to 1. Four possible results are shown. Red dot: Too positive expectations but too much uncertainty; this constellation is not considered as innovation. Mixed colour dots: The green/yellow innovation is ranked higher than the red/yellow innovation because less positive expectation combined with less uncertainty is higher ranked than more positive expectation combined with more uncertainty. Green dot: the combination of positive expectation with low uncertainty is considered as innovation. T: threshold.

These three components can be allocated to two dimensions which we believe are appropriate to classify innovations. The two dimensions concern the positive perspective and the uncertainty about expected risks and benefits (Fig. 1).

The positive perspective, expressing the power of motivation [13-15], is the most important criterion of innovation. An innovation is attractive if it offers the prospective of a solution to a hitherto unsolved problem. The greater the belief that the new innovation will offer benefit, the greater will be the demand for the promised solution. Not infrequently however, the need and desire to offer a new therapeutic principle will motivate the use of interventions that are not accompanied by convincing data.

On several occasions it is impossible to separate placebo effects [13] on one hand from the desired increase in motivation i.e. directing the patient towards the attainment of her/his goal [15]. A simple experiment performed at the Massachusetts Institute of Technology [16] helps illustrate this point. Students were randomly assigned to be informed that they had received an expensive painkiller or a cheaper version of the same painkiller. All of the subjects then received the same level of electric shock. Those told they had received the expensive medication perceived the shock as considerably weaker than their peers who believed they had taken the cheaper pill. Since both groups had actually been given placebos, this experiment confirms that the reality we perceive is substantially influenced by information and value judgments (e.g., expensive = better). The ethical evaluation of this experiment could influence its acceptance. We got the impression that colleagues who considered this experiment to be ethically problematic evaluated the information gained as less important than colleagues who accepted the ethical problem involved in this experiment. Those colleagues who accepted the terms of the experiment assume that the gain in information about the power of the placebo effect compensates the ethical problem because many incorrect conclusions could be avoided by this information, and risks to patients could thereby be reduced.

In summary, interest in an innovation increases the chances that the innovation will be evaluated positively. Rational considerations predict that the information, which induces a positive perspective, may not be evidence-based and may often lack validity. Neither the doctor who generates the positive perspective nor the patient who requests the positive perspective will be motivated to seek objective information that may discredit their faith in the innovation. Skepticism about the value of the innovation would destroy its power to mediate the positive perspective.

The second dimension of innovation is related to uncertainty of the results. In principle, it is relatively irrelevant whether the uncertainty is due to insufficient validity of the data or variability of the results. The important point is that only few reliable data are available at the time when a decision to either accept or reject an innovation must be made. In this case an attempt is made to use the few reliable data to support a statement that represents the best knowledge at the time the decision is being made

### Description of Steps in Innovation

Since innovations in prevention, diagnosis, and therapy cannot be evaluated according to identical criteria, for the sake of clarity we are listing them separately, even if that inevitably leads to repetition. The individual steps in innovation differ in the information available to support its benefit and are different in preventive (Table 1), diagnostic (Table 2) and therapeutic innovations (Table 3).

**Table 1:** Description of the steps in preventive innovations with examples.

**Step**	**Preventive innovation**	**Example**
I	**Preliminary data **confirm the optimization of the application of a preventive measure.	Improved tolerance, lower cost, or easier application of preventive measures.

II	**Preventive benefit **can be expected under **everyday conditions **due to high acceptance of the preventive measure.	High level of participation in the prevention program.

III	**Preventive benefit **can be expected under **everyday conditions **due to the proof **surrogate parameters **which are not only temporary.	Clinical results are achieved which hitherto could not be achieved without this prevention program.

**Table 2:** Description of the steps in diagnostic innovations with examples.

**Step**	**Diagnostic innovation**	**Example**
I	**Preliminary data **confirm an improvement in the feasibility or the results of diagnostic procedures (likelihood radio) in **clinical studies**.	Improved tolerance, easier application, or improved result of a diagnostic pro-cedure^a^.

II	**Diagnostic benefit **can be expected under **everyday conditions **due to the influence of the test result on the strategy of healthcare provision^b^.	It will be sufficient if the new diagnostic procedure enables a new therapeutic approach (independent of the success of this therapy).

III	**Diagnostic benefit **is confirmed under **everyday conditions **if a patient group can be identified that profits more than other groups from an available therapy.	The diagnostic innovation will be valuable if it enables a new therapy **and **this new therapy will be effective e.g. it improves QoL.

^a^Uncontested improvement in diagnosis does not guarantee improvement in treatment results. The quality of healthcare provision can be lowered by a growing gap between diagnostic and therapeutic possibilities. For this reason, isolated diagnostic successes are evaluated with caution. ^b^Influence on the strategy of healthcare provision means that the test result either influences the further diagnostic strategy or crosses the threshold from diagnosis to therapy. Influence on diagnostic strategy is confirmed if a positive test result leads to different diagnostic procedures than a negative test result.

**Table 3:** Description of the steps in therapeutic innovations with examples.

**Step**	**Therapeutic innovation**	**Example**
I	**Preliminary data **confirm optimized application of a therapeutic procedure. Only data on effect and safety from **clinical studies **are available.	Improved tolerance, lower cost, or easier application of therapeutic procedures.

II	**Therapeutic benefit **can be expected under **everyday conditions **due to a weak correlation of surrogate parameters^a ^with desired endpoints.	Reduction of tumor size after chemotherapy with unknown influence on quality of life or survival.

III	**Therapeutic benefit **is confirmed under **everyday conditions **by surrogate parameters which correlate highly with the desired endpoint(s) or achievement of endpoints.	Improvement in a defined area of quality of life or survival is confirmed.

^a^If surrogate parameters correlate only weakly, there is only a weak correlation between the confirmation of those surrogate parameters and the actually desired endpoint(s).

• In the first step of innovation, only data on the efficacy and on the feasibility of a measure would be required.

• The second step would require confirmation of the benefit of the innovation with surrogate parameters which may only weakly correlate with the desired endpoints.

• The third step, would require a high correlation between the surrogate parameters and the desired endpoints, i.e. evidence that the benefit of the innovation is sufficiently probable to justify public financing.

### Financing Innovations

Justification for public financing of an innovation would require the steps outlines in Table 4. This evidence would be reviewed by the impartial public agency described above.

**Table 4:** Proposed model for agreement on goal and limited financial support of innovations

**Step**	**Limited financial support**	**Agreement on goal**
I	Partial financing by risk sharing between the manufacturer and public fund for innovations.	The partners agree to strive for partial financing by private insurer and public fund.

II	Partial financing by private insurer and public fund for innovations.	The partners agree to strive for financing by a public fund for innovation.

III	Financing by public fund for innovations.	Elimination of remaining uncertain results by enlarging the database.

After agreement has been reached on specific goals, innovations which have not yet been included in publicly-financed healthcare are financed by a public fund for innovations for a defined amount of time. Depending on the extent to which proposed goals have been achieved, inclusion in publicly-financed healthcare provision will be determined at the end of the period of financing from the innovation fund.

Assessment and support of innovations increases the efficiency of a healthcare system because resources would be directed toward those innovations that have the highest probability of producing benefit. This leads to a desired redistribution of the available resources. When evaluating innovations, the break even point, which is considered appropriate by society, must be identified. This is the threshold that identifies levels of benefit that the public is willing to pay for [17].

The program would require the creation of a special set aside fund to finance higher risk innovations. Partial financing could provide important information about the willingness of the insured to pay, and a comparative value judgment could be obtained. However, this information can only be gained at the expense of at least temporary, unequal access to this innovation. It is also possible to obtain information concerning willingness to pay on the part of healthcare providers. A risk-sharing model oriented to the expected successes of the innovation could be applied here. For reasons of fairness in decision making, either both forms of information gain or neither should be accepted.

In the interest of competition, the extent of financing cannot be determined prospectively. It will depend on the amount of available financial resources, the quality of the innovation, the intensity of the demand, and the willingness of the public to pay.

### Practical implications

The steps in innovation describe the phases of information gain concerning the benefit of an innovation. In the beginning of these phases the only data available concern the effectiveness and the safety of the innovation. Data on benefit are typically not available. Safety and efficacy can justify licensure of an innovation but may not be enough to endorse public funding. Whether or not the innovation should be financed from public funds can only be decided when its benefit can be confirmed by a continuous gain in information. The goal of the innovation scale is to bridge this interval of indecision. It should ensure that promising innovations are available to all citizens, but remain publicly affordable.

Whether we approve of competition or not, stakeholders will decide which innovation prevails when the service of a different provider on the international market is better and more economical than that which the domestic provider can offer. Stakeholders can be the patients or purchasers of goods and services [18]. Technical and commercial services will be evaluated according to different quality criteria than services which place high demands on communication and the supply of specialist competence. It is, therefore, appropriate to work out criteria to assess the value of healthcare services and to place these in relation to costs [19]. Teleradiology and telepathology are services for which it should be relatively easy to develop uniform quality criteria for innovations.

It will be considerably more difficult to determine which methods should be used to decide whether something new should be recognized as an innovation. One recent debate considered whether the NICE Health Technology Assessment of therapy of the attention deficit/hyperactivity disorder (ADHD) met the demands of a well-balanced evaluation because NICE's economic model is dominated by cost differences [20,21]. This decision is relatively simple in comparison to the questions of why quantitative research is preferred to qualitative research and the techniques are subject to a hierarchic value judgment [22]. We know from experience that cultural differences, whether evidence based or not, are reality to patients, families and physicians. This is also true for innovations and the differences in recognizing them in different cultural circles. We need more discussion of criteria applied in different cultures to make decisions concerning the allocation of resources for innovations.

Finally, even a qualitative assessment of innovative healthcare services cannot conceal that successful reorganization of the system, which is often necessary for the implementation of innovations, involves the coordination and management of a complex process and not merely the realization of an isolated project [23].

## Summary

Quantitative assessment of innovations may be a helpful step for economic decisions about innovations

We propose a three step model based on the best available information at different developmental stages of the innovation evaluation process

Decision about reimbursement should be based on reviews by an impartial outcomes assessment agency

A public fund should be established to support evidence-based innovations.

An example is presented to illustrate how risk sharing models between manufacturers, private insurers, and the public fund can be adapted as new data on efficiency and effectiveness become available.

## Competing interests

The authors declare that they have no competing interests.

## Authors' contributions

Any of the authors contributed his essential part to this common product.

## Pre-publication history

The pre-publication history for this paper can be accessed here:


